# Reply: Artificial intelligence as a door opener for a new era of human reproduction

**DOI:** 10.1093/hropen/hoad045

**Published:** 2023-11-25

**Authors:** F Horta, M Salih, C Austin, R Warty, V Smith, D L Rolnik, S Reddy, H Rezatofighi, B Vollenhoven

**Affiliations:** Department of Obstetrics and Gynaecology, Monash University, Clayton, VIC, Australia; Monash Data Future Institute, Monash University, Clayton, VIC, Australia; City Fertility, Melbourne, VIC, Australia; Department of Obstetrics and Gynaecology, Monash University, Clayton, VIC, Australia; Department of Data Science and Artificial Intelligence, Faculty of Information Technology, Monash University, Clayton, VIC, Australia; Department of Obstetrics and Gynaecology, Monash University, Clayton, VIC, Australia; Department of Data Science and Artificial Intelligence, Faculty of Information Technology, Monash University, Clayton, VIC, Australia; Department of Obstetrics and Gynaecology, Monash University, Clayton, VIC, Australia; Department of Obstetrics and Gynaecology, Monash University, Clayton, VIC, Australia; Department of Obstetrics and Gynaecology, Monash University, Clayton, VIC, Australia; Women’s and Newborn Program, Monash Health, Melbourne, VIC, Australia; School of Medicine, Deakin University, Geelong, VIC, Australia; Monash Data Future Institute, Monash University, Clayton, VIC, Australia; Department of Data Science and Artificial Intelligence, Faculty of Information Technology, Monash University, Clayton, VIC, Australia; Department of Obstetrics and Gynaecology, Monash University, Clayton, VIC, Australia; Women’s and Newborn Program, Monash Health, Melbourne, VIC, Australia

Sir,

In response to the letter sent by Hengstschläger (2023) highlighting the importance of non-invasive multi-parameter embryo assessments and invasive multi-omics applications to enhance the low success rates of ART, we would like to discuss the main points raised.

First, although the introduction of simultaneous multi-parameter tools for non-invasive embryo assessments as well as invasive multi-omics applications lays significant promise in the field of ART, it is quite likely that the first beneficiaries of such technologies will be patients presenting multiple embryos to choose from, as a higher number of embryos reduce the risk of selection bias (Kragh and Karstoft, 2021) and facilitates subsequent embryo transfers (Ma et al., 2022). However, it is also likely that this is just the ‘tip of the iceberg’, considering the significant potential for multi-parameter analysis and emerging non-invasive technologies.

As described in Salih et al. (2023), artificial intelligence (AI) models have the potential to provide a tailored approach that is specific to the patients and their needs for a more personalized treatment by considering multiple variables, including cases of poor prognosis. For instance, under circumstances such as advanced reproductive age, traditional embryo selection criteria probably have less influence on the final reproductive outcomes, as the vast majority of the embryos produced by those patients are likely to be non-viable (Sanchez et al., 2019); thus, different strategies such as an alternative number of embryo transfers or fresh/frozen embryo transfer could be also suggested by AI systems. Importantly, the current utilization of poor-quality embryos is controversial, as low-grade embryos still possess the potential to result in a live birth (Kemper et al., 2021), whilst a ‘good’ quality embryo may not successfully implant (Wintner et al., 2017) even in the best or less favourable clinical circumstances. Consequently, future incorporation of multi-parametric information into AI models could aid in the further development of clinical decision support tools tailored to each patient's needs, including patients of poor prognosis presenting with a low number of eggs or embryos.

Nevertheless, it is crucial to highlight that the use of AI, even with the inclusion of multi-parameter analysis, does not universally enhance the efficacy of ART treatments. Currently, we are unable to improve the quality of gametes and the embryos produced through ART, despite knowing that gamete and embryo quality has a fundamental impact on reproductive outcomes (Vermey et al., 2019).

Considering this, there are two important aspects that could lead to improved outcomes even in cases of poor prognosis in alignment with the use of AI: (i) improving the understanding of gamete and embryo quality by non-invasive technologies and (ii) enhancing both the reproductive health of individuals and *in vitro* culture systems in ART.

i) The use of non-invasive technologies to monitor gamete and embryo health could facilitate a deeper understanding of the true meaning of ‘good’ and ‘poor’ quality by identifying the right non-invasive biomarkers.

For instance, remarkable studies by Shah et al. (2022) and Tan et al. (2022) have demonstrated that the metabolic activity of embryos can be non-invasively measured through metabolic imaging. Furthermore, aneuploid embryos exhibit distinct metabolic activity patterns that are detectable through metabolic imaging (Shah et al., 2022; Tan et al., 2022). Substantial progress has been made in ploidy prediction using AI and metabolic imaging (Tan et al., 2022). Moreover, it is worth noting that even embryos labelled as ‘good’ quality can display abnormal metabolic activity indicative of aneuploidy during non-invasive measurements (Shah et al., 2022).

In the future, approaches incorporating metabolic imaging and AI could pave the way for selecting the highest-quality gametes and embryos based on their metabolic activity. Identifying the metabolic patterns of ‘truly good’ quality embryos non-invasively could facilitate the development of novel AI monitoring systems, thereby enabling the exploration of alternative strategies to enhance gamete and embryo quality, especially in cases with poor prognosis.

ii) Enhancing both the reproductive health of individuals and *in vitro* culture systems necessitates novel multi-parameter assessments where AI could play a crucial role in monitoring and ensuring safe application.

Outstanding work by Bertoldo et al. (2020) has demonstrated the potential for improving oocyte and embryo quality particularly in cases of poor prognosis, where both gamete and embryo quality may be compromised due to advanced reproductive age. Substantial research has also been conducted on the use of antioxidants to enhance reproductive health in individuals as well as *in vitro* culture systems (Zarbakhsh, 2021).

However, future efforts aimed at enhancing the overall reproductive status of both individuals and their gametes/embryos require the initial clarification of the true meanings of optimal reproductive health and ‘good’ quality, especially in cases involving gametes and embryos. Given the complexity of the variables that contribute to this definition, the use of AI for multi-parameter assessments can aid in identifying what constitutes optimal reproductive health. This approach would enable us to monitor whether novel treatments genuinely improve reproductive health or, alternatively, identify the optimal responses tailored to the individual circumstances of each patient as well as their gametes and embryos.

Finally, without a doubt, it is crucial that, for the benefit of both patients and clinicians, substantial ethical research is conducted, from the bench to clinical practice, to assess the convergence of these technologies into a new era for ART.
